# X-ray phase-contrast imaging at 100 keV on a conventional source

**DOI:** 10.1038/srep05198

**Published:** 2014-06-06

**Authors:** T. Thüring, M. Abis, Z. Wang, C. David, M. Stampanoni

**Affiliations:** 1 Paul Scherrer Institute, Villigen PSI, Switzerland; 2 Institute for Biomedical Engineering, Swiss Federal Institute of Technology, Zurich, Switzerland

## Abstract

X-ray grating interferometry is a promising imaging technique sensitive to attenuation, refraction and scattering of the radiation. Applications of this technique in the energy range between 80 and 150 keV pose severe technical challenges, and are still mostly unexplored. Phase-contrast X-ray imaging at such high energies is of relevant scientific and industrial interest, in particular for the investigation of strongly absorbing or thick materials as well as for medical imaging. Here we show the successful implementation of a Talbot-Lau interferometer operated at 100 keV using a conventional X-ray tube and a compact geometry, with a total length of 54 cm. We present the edge-on illumination of the gratings in order to overcome the current fabrication limits. Finally, the curved structures match the beam divergence and allow a large field of view on a short and efficient setup.

X-ray radiography and computed tomography (CT) are standard imaging techniques in materials and life sciences for the nondestructive examination of samples or diagnostic tasks in medicine. The underlying contrast mechanism relies on the different X-ray attenuation properties of different materials or tissue types. The dominant physical effects contributing to attenuation are the photoelectric effect and incoherent (Compton) scattering. Besides attenuation, the wave nature of X-rays reveals another contrast mechanism, which is the phase shift. The interaction contributing to phase shifts is coherent (Rayleigh) scattering^1^.

The attenuation and phase shift properties are described by the complex refractive index *n* = 1 − *δ* + *iβ*. The imaginary part *β* is related to the attenuation coefficient by *µ* = 4*πβ*(*λ*)/*λ*, while the real part *δ*(*λ*) determines the phase shift *ϕ* = 2*πδ*(*λ*)/*λ*.

While the attenuation can be measured with an X-ray detector as the reduction of the beam intensity, the phase is not directly observable. Therefore, an optical system is needed to convert the phase shift into intensity modulations.

Phase-sensitive imaging is a desirable modality, since it delivers a complementary source of contrast with respect to absorption by providing direct access to the electron density^1^. Moreover, the combination with attenuation enables the determination of the effective atomic number^2^. An enhanced contrast-to-noise ratio (CNR) compared to attenuation for certain materials or tissues^3^,^4^ has also been demonstrated.

The vast majority of phase-sensitive techniques, including crystal analyzer based^5^,^6^ or interferometric^7^,^8^ methods rely on X-ray beams of high spatial and temporal coherence, which are available only at synchrotron sources. Inline phase contrast^9^,^10^,^11^ and Talbot interferometry^12^,^13^,^14^ need high spatial coherence but are available on polychromatic microfocus sources. Phase-contrast imaging using X-ray beams of low temporal *and* spatial coherence such as conventional low-brilliance X-ray tubes have been demonstrated with coded apertures^15^ and Talbot-Lau interferometry^16^. Analyzer-based systems have been recently extended to tube sources^17^,^18^ but only at energies up to the tungsten *K_α_* line at 60 keV. In addition to phase sensitivity, crystal analyzers and Talbot interferometers also provide — with different retrieval mechanisms — information about the integrated local small angle scattering power from microscopic density fluctuations in a specimen^19^. This signal is known under the name of dark-field, scatter or visibility reduction contrast.

High-energy Talbot interferometry has been reported so far using a synchrotron source at nominal energies of 82 keV^20^. Using a low-brilliance X-ray tube, Talbot-Lau interferometry was applied so far at 60 keV design energy^21^. Medical imaging applications may benefit from phase contrast at higher energies: chest or abdominal radiography or CT require an acceleration voltage between 100 and 150 kVp. Other potential applications are homeland security or chip failure analysis, which require high energies for the visualization of materials of high density and atomic number.

## Results

We introduce a method for phase contrast imaging which works on conventional X-ray sources, covers the entire diagnostic X-ray energy range and is compatible with compact imaging arrangements. The approach is based on Talbot-Lau interferometry^16^ and employs an edge-on illumination approach for the grating design and arrangement. Our solution removes one of the major hurdles which prevented grating interferometry from being applied at high energies so far, namely the fabrication of gratings with high aspect ratios. The aspect ratio, given by 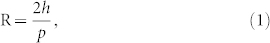
where *p* is the grating period and *h* the structure height, is limited by the fabrication process, usually photolithography^13^ or X-ray lithography^22^ since grating structures tend to collapse or deform (e.g. due to capillary forces) if the aspect ratio is too high. Moreover, when using a broad spectrum, photons above the design energy should also be efficiently blocked by the gratings, thus requiring even higher aspect ratios. The largest aspect ratios achieved by current fabrication techniques^23^,^24^ are around 60. For a given setup length these parameters depend on the target energy *E* according to 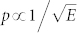 and *h* ∝ *E*^3^, and therefore R ∝ *E*^7/2^ 14. If at *E* = 25 keV an aspect ratio for the absorption grating around R = 30 is sufficient, it would have to be at least 128 for *E* = 100 keV. With our design, we reach an aspect ratio of 143, satisfying the above condition with a transmission of less than 1% at 100 keV. As a comparison, a recent interferometer implemented at a third generation synchrotron facility^20^ reported an aspect ratio of 21 with a transmission as large as 20% at 82 keV.

**Figure 1:**
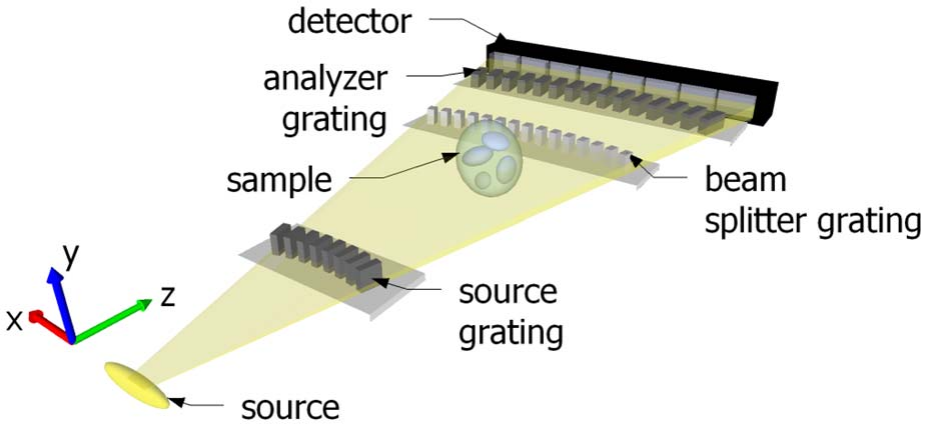
Schematic of a grating interferometer for X-ray energies between 80 and 150 keV in edge-on illumination mode. The aspect ratio is defined by the ratio of the traveling distance along the grating lines and the period and can be arbitrarily long. In order to maximize the field of view, the grating structures are aligned on an arc. A 100 keV setup was realized where the distance between the source and the source grating is 23 cm and the distance between the source grating and the phase grating is 16 cm. That is also the distance from the phase grating to the analyzer grating.

Our design introduces edge-on illuminated, circularly aligned structures. Edge-on illumination (Fig. 1), as opposed to face-on illumination, exploits the dimension along the grating lines to form a high aspect ratio of the structures in the direction of the beam^34^. The effective structure height of the grating is then determined by the grating dimension along the grating lines, which essentially allows arbitrarily high aspect ratios.

Increasing the aspect ratio of the gratings typically leads to a reduction of the field of view due to the change of the grating transmission function at high incident angles. In order to overcome this problem, the grating lines are circularly aligned with a radius equal to the distance to the source. This allows to achieve an arbitrarily large field of view in a fan-beam geometry, a significant improvement compared to face-on based and glancing angle^25^ approaches.

The combination of edge-on illumination and circularly aligned structures enables phase-contrast imaging at arbitrary design energies and with a maximum field of view in the horizontal direction (*x* direction). These advantages come at the expense of a limited field of view in the vertical direction (*y* direction), which is, depending on the X-ray detector, typically a few pixels. However, radiographic 2D imaging can be obtained by scanning the sample or a thin fan beam. The scanning technique has been demonstrated to deliver less dose than the conventional approach based on the illumination of a large area. In digital mammography, for instance, where dose is a critical issue, Philips' MicroDose system combines a scanning approach with a highly collimated fan beam^26^. Thanks to the high collimation, the dose deposited on patients has been reported to be significantly lower than with other instruments based on the illumination of a large area detector^27^. Similarly, for tomographic images, the approach allows single slice CT or full 3D imaging in scanning mode.

**Figure 2:**
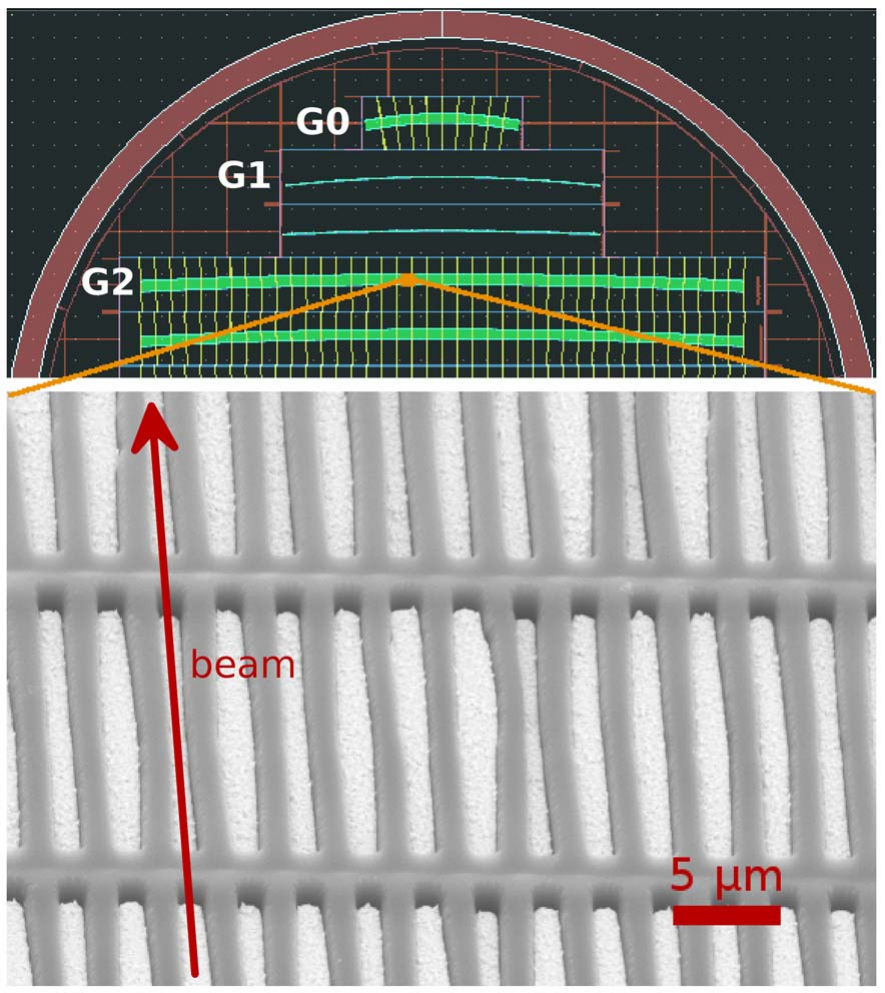
Grating design mask for the edge-on illumination approach and SEM image of the grating. The top part of the 4 inch wafer shows five grating chips. From top to bottom, one source grating, two phase gratings and two analyzer gratings. The gratings have different curvatures which are specific to the grating interferometer geometry. Multiple gratings for more than one setup geometry are fabricated on a single wafer. The SEM image shows the gold structures and the interrupting bridges that prevent the lamellae from collapsing^24^.

Grating design and fabrication is nonstandard and involves a complex mask design, as shown in Fig. 2. Multiple gratings can reside on a silicon chip with their specific structure length and curvature. For the current experiments, a symmetric interferometer with a grating period of *p* = 2.8 µm for all gratings has been used. The design energy is 100 keV and the beam splitter grating periodically shifts the phase by zero and *π* at this energy^13^. Using gold as the phase shifting material, a structure length of *h*_1_ = 19.8 µm is required. The analyzer grating is an absorption mask for sensing slight changes of the interference pattern generated by the beam splitter^14^. With a structure length of *h*_2_ = 800 µm it can absorb more than 90% of the incoming X-rays up to energies of 160 keV. The beam splitter and analyzer grating are separated at the first fractional Talbot order^28^, resulting in an intergrating distance of 158 mm. However, the precise position of the analyzer grating along the beam axis is not critical, and a change in visibility of less than 1% is observed by displacing it by as much as 5 mm. The source grating splits the relatively large focal spot (~ 1 mm) into an array of individually coherent, but mutually incoherent sources^16^. It is also made of gold structures with a length of *h*_0_ = *h*_2_ = 800 µm.

Due to the large spectral acceptance^28^,^29^ of the interferometer (50 keV to more than 160 keV) and the high attenuation efficiencies of the source and analyzer gratings (more than 90% up to 160 keV), the voltage of the X-ray source was set to the maximum of 160 kV. With a structure height of approximately 100 µm, the field of view in the vertical direction is limited to one detector pixel row. In the horizontal direction, the field of view is only limited by the grating size to 30 mm, but wider gratings can be fabricated with the same method and the available technology on larger wafers. In addition to the standard components (source, camera, interferometer), two optical slits, one in front of the source grating, the other in front of the camera, were required for the collimation of the beam in the vertical direction.

**Figure 3:**
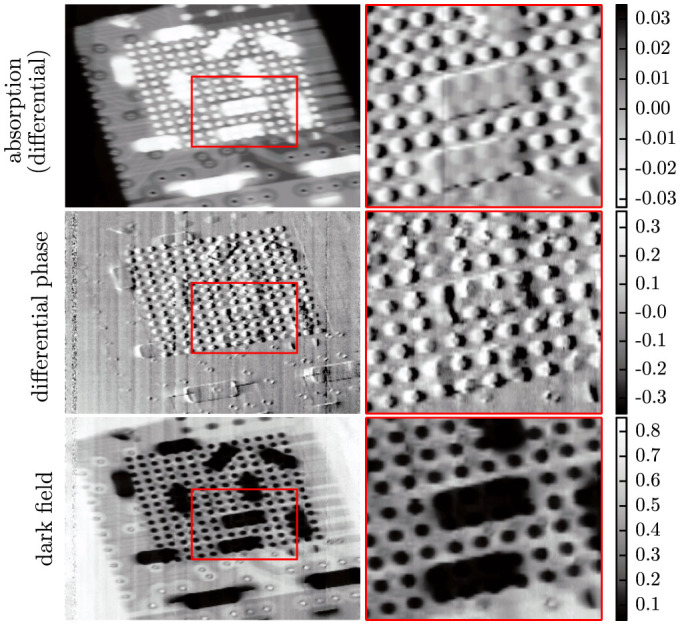
Radiographic scan of an electronic chip. The image was acquired with 24 phase steps per line and an exposure time of 15 s per step. The top right image shows the differential absorption image.

Fig. 3 shows the first X-ray images acquired with our new setup, based on edge-on illuminated grating interferometry powered by a conventional X-ray source operated at 160 kVp and with a design energy of 100 keV. Several resistors and integrated circuits located on different layers on an electronic chip can be identified. The radiographs were acquired in scanning mode, using a step size of 100 µm along the *y* axis, covering a total imaging area of 2 × 2 cm^2^. For a better comparison of the magnified phase and attenuation images, the latter has been replaced with the differential attenuation image, which was obtained by calculating the derivative along the horizontal axis. In the attenuation image, the contrast of the soldering points of the integrated circuit is reduced underneath the resistors, while in the phase image, they can clearly be identified. The reduced contrast of the soldering points in the absorption image is due to beam hardening. The spectrum impinging on these soldering point is hardened by the resistors in the upper layer, resulting in lower absorption contrast. Due to the weaker energy dependence of phase shifts (1/*E* compared to 1/*E*^3^), phase-contrast images are less sensitive to beam hardening^30^, which explains the lower contrast reduction of the soldering points under the resistors in the phase image of the chip. This example shows one of the potential benefits of phase contrast X-ray imaging at high energies, which may be useful to identify flaws in multilayered structures such as electronic chips.

## Discussion

Possible usages of edge-on grating interferometry encompass applications ranging from medical imaging to non-destructive testing and homeland security. All these applications seek for high (greater than 100 keV) nominal energies and compact designs. Particularly for medical application, the operation of a phase contrast imaging device at high energies is imperative. When imaging patients, a crucial issue is the dose, which depends mainly on the absorption of the X-rays and decreases with the third power of the photon energy^31^. On the other hand, the phase interaction is only inversely proportional to the energy and is therefore relatively much more relevant than absorption as the energy increases, possibly leading to a decreased exposure for an equivalent image quality. Clinical operation of such a device will imply measuring patients who normally breath, move and shake. Fast acquisitions are therefore required, in order to keep the images sharp and artifact free. As a consequence, compact, efficient imaging systems need to be developed to provide imaging capabilities at sufficient speed. Our circular grating alignment matches the structures of the diffracting and absorbing elements to the divergent beam, especially for compact geometries. In conclusion, short, high-energy phase-contrast systems will enable the efficient investigation of high-density materials or thick samples, adding information on electron density and integrated small-angle scattering power to the conventional absorption-based signal. This will improve material discrimination and density sensitivity in future X-ray or even neutron^32^ investigations.

## Methods

Edge-on illuminated gratings were manufactured by Microworks GmbH, Germany, using a LIGA process^24^. Each grating resides on a 5 × 60 mm^2^ silicon chip and several grating chips are fabricated on a single 4 inch silicon wafer. The experimental arrangement for a design energy at 100 keV is a symmetric Talbot-Lau interferometer with a grating period of *p* = 2.8 µm for all gratings. The distance from the source grating to the analyzer grating is 32 cm and the source grating is positioned 23 cm away from the source.

The X-ray source is a tungsten target COMET MXR-160HP/11 X-ray tube with a maximum output voltage of 160 kV and a current of 10 mA. In the experiment, it was set to the maximum voltage. The focal spot size is approximately 1 mm. The detector is a CCD camera from Finger Lakes Instruments. A cesium iodide (CsI:Tl) scintillator of 600 µm thickness converts the X-rays to visible light and is coupled to an optical lens projecting the image onto the CCD. The effective pixel size is 80 µm. The collimating slit right after the source is 25 µm wide, while the second slit before the detector is 100 µm.

In Fig. 3, image acquisition involved 24 phase steps per line and an exposure time of 15 seconds per step. The long exposure times are mostly constrained by the low average visibility of the gratings (5%). The exposure time was chosen in order to get a low noise in the differential phase image. The signal-to-noise ratio (SNR) is proportional to the visibility and the square root of the exposure time^33^. This implies that the exposure times can easily drop by an order of magnitude as these gratings become comparable in quality to those developed in the last ten years. Smaller regions of these gratings already exhibit a visibility up to 14%, indicating that this goal is reachable as the fabrication becomes more reliable and uniform.

## Author Contributions

C.D. and M.S. conceived the edge-on illumination scheme for grating interferometry. T.T., Z.W. and M.A. designed the first experiment. T.T. and M.A. performed the experiment and analyzed the data. T.T. and M.A. wrote the manuscript with contributions from all authors.
